# Resistant social anxiety disorder response to Escitalopram

**DOI:** 10.1186/1745-0179-2-35

**Published:** 2006-12-13

**Authors:** Stefano Pallanti, Leonardo Quercioli

**Affiliations:** 1 Mount Sinai School of Medicine, New York, USA; 2 Assistant Psychiatrist, Istituto di Neuroscienze, Florence, Italy; 3 Department of Neurological and Psychiatric Sciences, University of Florence, Italy; 4 Istituto di Neuroscienze, Viale Ugo Bassi 1, 50137 Florence, Italy

## Abstract

**Background:**

Social Anxiety Disorder (SAD) is a common disorder and its high prevalence and lifelong chronicity are such that it represents a substantial public health problem. The observation that serotonergic agents appear to be effective for its treatment suggests that patients may have abnormal serotonergic neurotransmission within the central nervous system. We investigated the efficacy of Escitalopram in treatment resistant patients with SAD.

**Method:**

Twenty-nine adult outpatients participated in a 12-week open-label trial of escitalopram. All the subjects had a primary diagnosis of SAD and had failed at least one previous adequate trial of paroxetine. Escitalopram was orally administered starting with a dose of 10 mg/day following a 1-week titration.

**Results:**

The escitalopram treatment was characterized by good tolerability (drop-out rate due to intolerance: 10.3%), and 24 subjects completed the study trial. At the end of the 12-week treatment period, 14 subjects (48.3%) were considered as responders on the basis of the Clinical Global Impression-Improvement (CGI-I) (much or very much improved) scale and the Liebowitz Scale for Social Anxiety (LSAS) (reduction >35% compared to baseline).

We observed a significant mean reduction in the Sheehan Disability Scale Work (p < .05) and Social (p < .05) subscores, but not in the Family subscore.

**Conclusion:**

These data suggest escitalopram has a role in the treatment of resistant SAD, especially in view of the favourable tolerability profile observed in the patients. Controlled studies are required to further investigate these findings and to compare escitalopram with other treatments for this disorder.

## Background

Selective serotonin reuptake inhibitors (SSRIs) are being used more frequently as treatment for generalized and specific Social Anxiety Disorder (SAD), a common illness with significant associated disability. Estimates of lifetime prevalence in adult samples, when modern diagnostic criteria are used, range from 3.9% to 13.7% [1,2]. The prevalence estimates are generally higher in women than in men.

Individuals with SAD are more likely to develop disabilities in the areas of school, work, and social life, with particular problems initiating relationships with the opposite sex. Furthermore, increased disability and a reduced quality of life, as well as increasing rates of comorbidity with secondary mental disorders (e.g., depression, substance abuse) can be expected to develop over time [3].

The stability of the SSRI effect size estimate in conjunction with other evidence for safety and tolerability, and the ability of SSRIs to treat comorbid conditions, support their use as a first-line treatment [4,5].

More specifically, paroxetine was the first SSRI indicated for the treatment of SAD, and its efficacy in the treatment of acute social anxiety disorder has been clearly demonstrated in four 12-week, randomized, placebo-controlled studies [6-9]. More recently, paroxetine's effectiveness in the long-term treatment of SAD has also been shown [10].

However, about 50% of cases do not respond to a first SSRI treatment. Treatment resistance has been a topic of interest in many psychiatric disorders, but clear and standardized definitions of response, resistance and remission to treatment in SAD warrant further discussion and need to be based on empirical data [11].

The results of several studies suggest that citalopram may be a safe and effective treatment for SAD, including patients who have failed to tolerate or respond to a prior treatment trial [12-14]. A recent investigation showed that patients with SAD and healthy controls did not display differences to a citalopram challenge (20 mg over 30 min) in terms of 5-HT neuroendocrine response measured as increased plasma concentrations of prolactin and cortisol [15]. However the finding of an increased headache response in patients with SAD may indicate hypersensitivity of the serotonergic pathways related to some subtypes of 5-HT_2 _or 5-HT_3 _receptors following the citalopram administration [15].

Headache is a well-known side effect of serotonergic medications. The specific mechanism responsible for SSRI-induced headache is not clear and it is usually attributed to the agonistic effects on the post-synaptic 5-HT receptors. Citalopram is a racemic drug, and its effects on serotonin transport are thought to reside in the S-enantiomer, known as (S)-citalopram or escitalopram. Escitalopram is the most selective SSRI yet developed. Its receptor binding properties and activity in preclinical animal models of depression predict the effectiveness of escitalopram in the treatment of depression, with approximately twice the potency of the racemate [16,17].

Escitalopram has shown significant superiority to placebo in controlled clinical trials of depression, social anxiety disorder, generalized anxiety disorder, and anxiety symptoms associated with major depression [18-24]. In December 2003 escitalopram received FDA approval for the treatment of Generalized Anxiety Disorder.

The aim of the present study is to investigate the effectiveness and tolerability of escitalopram in a group of treatment-resistant patients with SAD.

## Methods

### A. Subjects

Twenty-nine adult outpatients at the Institute for Neurosciences in Florence (Italy) were consecutively enrolled in the study from January to June 2006. All the subjects met the DSM-IV criteria for the diagnosis of SAD, present for at last one year, and established by means of the structured clinical interview for DSM-IV (SCID-I, SCID-II). The SCID interviews were conducted by two psychiatrists (LQ, ARP) certified for the use of this instrument.

All the patients had previously failed at least one adequate trial of paroxetine treatment(>= 60 mg/d for >= 12 weeks). Paroxetine was the only labeled approved drug for SAD in Italy. Recently, also escitalopram has been approved in Italy for SAD treatment. "Failure" was defined as: 1. experiencing less than a 35% decrease from baseline in the Liebowitz Scale for Social Anxiety total score, and a score of 'minimal improvement' or less on the Clinical Global Improvement scale (CGI) after 12 weeks of treatment. The mean length of these failed trials was 18.1 weeks, sd 4.2, during which each patient had been pushed to the maximum tolerated medication dose; 2. having stopped the medication in the first 3 weeks of treatment because of intolerable side effects or lack of compliance.

Patients with a concurrent diagnosis of major depressive episode or marked depressive symptoms scoring >18 on the 21-item Hamilton Depression Rating Scale (HDRS) were excluded from the study. Other exclusion criteria were: a concurrent Axis-I diagnosis (DSM-IV) for schizophrenia or other psychotic syndromes; any form of substance dependence (active in the last year) or any substance abuse including alcohol within the last three months; Tourette syndrome or other tic disorders, Bipolar-I disorder, organic brain syndromes, pregnancy, nursing, active suicidal thoughts or serious suicide risk. Ten of the 29 subjects (34.5%) reported having failed to respond satisfactorily to an adequate period (at least 16 consecutive appointments) of cognitive psychotherapy. No cognitive or behavioral psychotherapy was allowed during the treatment and follow-up period.

After receiving an explanation of the potential risks and benefits of the escitalopram treatment and of alternative treatments, each patient gave written informed consent in accordance with the Declaration of Helsinki.

### B. Protocol

The study was a 12-week open trial of escitalopram treatment. After this period, patients who showed at least moderate response to treatment (>= 35% reduction of LSAS total score) continued open treatment with escitalopram at the maximal received dose until day 84. Patients who showed no change after the 12-week period were offered a co-treatment with a different SSRI, or MAOI was started.

The escitalopram treatment began after a 1-week (two weeks for fluoxetine) washout period. The escitalopram titration dosing was established as follows: 10 mg during days 1 to 7, 20 mg from day 8. It was established that the minimum tolerated citalopram dose for persisting in the study was 10 mg. Tablets were to be taken once a day in the morning. During the study period, the only permitted co-treatment for controlling sleep disorders was a low-dose benzodiazepine medication (lorazepam 1–2 mg, temazepam 20 mg daily).

### C Assessment

At baseline, all the patients were assessed with the SCID-I and SCID-II diagnostic interviews, the Liebowitz Scale for Social Anxiety (LSAS), the CGI-I and CGI-S (Clinical Global impression scales for -Improvement and -Severity), the Sheean Disability Scale (SDS), the Arizona Sexual Experience Scale (ASEX), and the Hamilton Depression Rating Scale (HDRS). The LSAS, the CGI-I and CGI-S, and the Dosage Record and Treatment Emergent Symptom Scale (DOTES) for side effects were administered at each assessment visit pre-planned at days 14-28-42-56-70-84. At day 28–56 and 84 the ASEX and the SDS were repeated. The LSAS and CGI-S were used as the primary efficacy variable. ASEX and SDS scales were considered as secondary efficacy indicators.

### D Statistical analysis

Mean values and SD and ranges were calculated for all parametric variables. Interrater reliability was ascertained through a series of live independent ratings by the authors (S.P., L.Q.) and yielded intraclass correlations of 0.88 for the HDRS (Cronbach α). Student t-tests for independent variables, ANOVA for repeated measures, Pearson-rho correlation coefficients were performed where appropriate, with alpha set at p < 0.05, two tailed. Data were analyzed using an SPSS-PC package, running on a Pentium-II PC compatible.

## Results

The demographic and clinical characteristics of the subjects are presented in Table 1. Of the 29 patients enrolled for the trial, 24 (82.7%) completed the 3-month period of treatment with Escitalopram. Three patients dropped out of the trial due to non-compliance without significant side effects, and two patients interrupted the trial in the first week of treatment for adverse effects, in particular increased anxiety/agitation. Other relatively common treatment-emergent side effects observed during the trial were nausea (20.7% cases), anxiety (17.2%), headache (17.2%), somnolence (10.3%), fatigue (6.9%) and diarrhea (6.9%).

**Table 1:** Demographic and clinical characteristics of the group of 29 patients with resistant Social Anxiety Disorder (SAD) enrolled in the study.

Age	m: 34.6 sd: 9.9 (range 24–42)	
Gender	13 M (44.8%)/16 F (55.2%)	
Education level (yrs)	m: 12.3 sd: 4.6	
Marital status	Single n = 9 (31%)	
	Married n = 16 (55.2%)	
	Divorced n = 4 (13.8%)	
Duration of SAD (yrs)	m: 12.9 sd: 7.3	
Baseline LSAS score	m: 62.4 sd: 10.6	
Baseline HSRD score	m: 12.68 sd: 2.42	
Baseline CGI-S	m: 4.8 sd: 1.1	
CGI-I (Paroxetine trial)	m: 4.3 sd: 0.5	
Baseline SDS Work	m: 7.6 sd: 1.8	
Baseline SDS Social	m: 8.5 sd: 2.0	
Baseline SDS Family	m: 5.2 sd: 1.3	
Baseline ASEX	m: 19.4 sd: 2.3	
Lifetime comorbidity		
*Axis I*	Depressive disorder	5 (17.2%)
	Cyclothimia	2 (6.9%)
	Panic disorder	6 (20.7%)
	Somatoform disorder	3 (10.3%)
	Obsessive-Compulsive Dis.	2 (6.9%)
	Alcohol abuse	5 (17.2%)
	Substance abuse	2 (6.9%)
*Axis 2*	Avoidant PD	4 (13.8%)
	Obsessive-Compulsive PD	1 (3.4%)
	Passive-Aggressive PD	1 (3.4%)
	Narcissistic PD	1 (3.4%)

Key:

LSAS: Liebowitz Scale for Social Anxiety.

HSRD: Hamilton Depression Rating Scale.

CGI-S: Clinical Global Inventory-Severity.

CGI-I: Clinical Global Inventory-Improvement.

SDS: Sheehan Disability Scale.

ASEX: Arizona Sexual Experience Scale.

At the end of the 12-week period of treatment, 2 patients were receiving escitalopram 10 mg/day, 3 patients 15 mg/day and 19 patients 20 mg/day. The mean maximum escitalopram dose received by subjects who completed the 12-week trial was 18.54 mg (sd: 3.12). The mean daily escitalopram dose received by each subject across the 12-week period was 16.22 mg (sd: 4.21).

**Figure 1:**
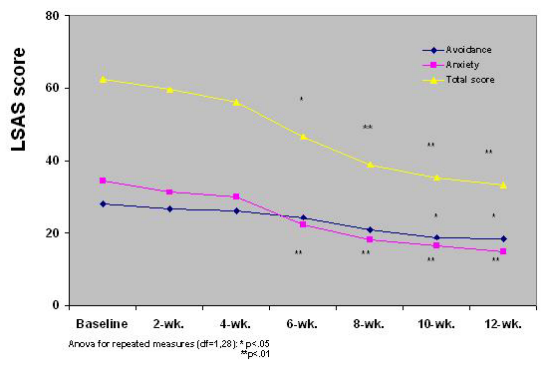
LSAS mean total and sub-scores in 29 resistant patients with SAD treated with a 12-week period of daily active escitalopram.

Compared to baseline, the mean LSAS total score dropped over the 12 weeks of treatment to 33.21 sd 6.12 (n = 29) (ANOVA-F repeated meas. = 9.62, df:1,28; p < .01) (see figure 1). The range of LSAS scores fell from 51–80 to 12–61. The mean reduction in total LSAS scores was -23.2 sd 8.3 (range +6/-52), which represented a percentage reduction of -37.18% sd 12.9 (range +12.7%/-66.6%). Of the subjects who completed the trial period, 14 (48.3%) patients experienced an LSAS total score decrease of >= 35%, 5 of them (17.2%) an LSAS score decrease of >= 50%. Depressive symptoms, expressed as a mean HSRD score, were not considered as an efficacy measure in the present study. However, they appeared to have reduced significantly after 3 weeks (end of the trial: m = 8.11, sd:3.05; ANOVA-F repeated meas.: 7.28, df:1.28 p < .03). The amelioration of the social phobia symptoms was reflected also by a mean reduction of CGI-S scores, which reached significance from the 6^th ^week of treatment (baseline: m: 4.8, sd: 1.1; end of trial: m: 2.2, sd:1.3, ANOVA-F repeated meas.: 9.37, df: 1.28, p < .01).

The maximal daily escitalopram dose showed a positive but not significant correlation with the reduction of the LSAS total score after 12 weeks (Pearson-ρ :.32, n = 29, n.s.). At the end of the trial we observed a significant reduction in the Sheehan Disability Scale Work (m: 5.3, sd: 1.7; ANOVA-F repeated meas.: 6.11, df: 1,28, p < .05) and Social subscores (m: 6.4, sd: 1.9; ANOVA-F repeated meas.: 6.81, df: 1,28, p < .05), but not in the Family subscore (m: 4.6, sd: 1.3; ANOVA-F repeated meas.: 3.19, df: 1,28, n.s), compared to the baseline.

**Figure 2:**
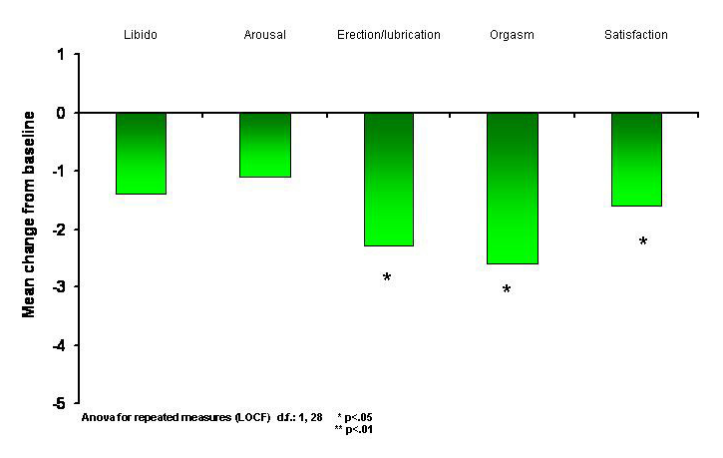
Mean variation on the ASEX subscale scores between baseline and end of trial (12^th ^week) in resistant social phobic patients treated with Escitalopram (n = 29) (score reduction corresponds to decreased side-effect).

After 12 weeks of treatment, social phobic patients showed a reduction of severity and pervasiveness of sexual side effects as reported through the ASEX scale. In particular, significant amelioration was found on the Erection/Lubrication (F = 6.74, df = 1,28; p < .05), Orgasm (F = 5.81, df = 1,28, p < .05), and Satisfaction (F = 6.55, p < .05) subscales (all df = 1, 28). No significant differences were found in the Libido and Arousal subscales (see figure 2)

## Discussion

The main result of the study is the significant mean reduction of LSAS scores and CGI-S scores (primary indicators) in resistant social phobic patients after a 12-week period of escitalopram treatment. A progressive reduction of LSAS scores was rated and reached significance from the 6^th ^week of treatment. The SDS scales indicated that clinical improvement corresponded to a significant reduction of social disability related to the disorder, at least in the work and social spheres. The LSAS Anxiety subscore decreased earlier and more significantly than the Avoidance subscore through to the 6^th ^week of treatment. This is in accordance with the observation that behavioral amelioration of SAD in response to treatment follows the reduction of psychological anxiety states related to social contexts. However, we were unable to ascertain whether subjects with resistant SAD are a homogeneous subsample amongst SAD patients. The main limit of the present study is its open-label design and the lack of a placebo group, so it is hard to say how much the placebo effect is responsible for the positive results. Nor does it account for experimenter bias. In fact, one of the limits of this study is that it did not consider the weight of single comorbidities, especially in resistant subjects, and their possible impact on the response to treatment. Furthermore, it cannot be excluded that other classes of drugs (e.g., anticonvulsants) might be useful in some resistant SAD subjects depending on the impact of the comorbid condition (e.g., bipolar spectrum disorders) on SAD. About 50% of the initially enrolled subjects were classified as responders to escitalopram. This percentage is consistent, taking into account that all the subjects were previously resistant to paroxetine treatment. While the effectiveness of escitalopram in SAD has already been reported, this is the first study of escitalopram treatment in subjects with resistant SAD [20]. The drop-out rate in the 3-month trial period was 17.3%, suggesting an average good tolerability and mild side effects profile for escitalopram in this group of subjects. The drop-out rate resembles that found by Lader et al, while it appears higher than that found by Kasper et al among patients with SAD treated with escitalopram [20,21]. More specifically, in our study escitalopram appeared more tolerable in terms of sexual side effects in comparison with paroxetine in the same subjects, as evidenced by the reduction of several ASEX subscales from baseline (under paroxetine treatment) and at the end of the trial (under escitalopram treatment). Lader et al found that the percentage of sexual side effects (ejaculation failure, libido decreased, impotence) associated with escitalopram 20 mg/day and paroxetine 20 mg/day in SAD were roughly similar [20]. It is of note that all our group of patients had previously failed an adequate trial of paroxetine treatment at dosage >= 60 mg/d for >= 12 weeks. It is also our clinical experience that a paroxetine dosage of 20 mg/day can be considered low for the treatment of SAD. We believe that the relatively low sexual side effects found by Lader et al using paroxetine was due to the sub-therapeutical dosage of this drug.

## Conclusion

Escitalopram extends the possibility of treating subjects with SAD who are resistant or intolerant to other SSRI or IMAO treatment. Given the safe side effects profile, the possibility of using it for non-resistant patients should also be considered. At this time we believe that this schedule may be useful in treating SAD non-responders.
